# Co-Valorization of Waste Cooking Oil and Expanded Polystyrene Pyrolysis Fractions as Potential Fuel Blendstocks

**DOI:** 10.3390/polym18111341

**Published:** 2026-05-28

**Authors:** Arantxa M. Gonzalez-Aguilar, James R. Vera-Rozo, José M. Riesco-Ávila

**Affiliations:** 1 Faculty of Mechanical Engineering and Naval Sciences, Veracruzana University, Veracruz 94294, Mexico; arantgonzalez@uv.mx; 2 Mechanical Engineering Program, University of Pamplona, Pamplona 543050, Colombia; james.vera@unipamplona.edu.co; 3 Mechanical Engineering Department, Engineering Division, Campus Irapuato-Salamanca, University of Guanajuato, Salamanca 36885, Mexico

**Keywords:** waste cooking oil, expanded polystyrene, pyrolysis oil, alkaline-assisted co-processing, fuel blendstock, circular economy

## Abstract

The energy demand, depletion of fossil fuels, generation of plastic waste, and final disposal of waste cooking oil (WCO) have become major concerns due to industrialization and population growth, creating significant environmental challenges. These challenges have encouraged the development of sustainable alternatives for the valorization of residual feedstocks. On the one hand, global energy consumption continues to increase, promoting the search for alternative fuel sources; on the other hand, the improper disposal of plastic waste has motivated the development of recycling technologies for plastic residues that are difficult to recycle through conventional routes. Moreover, WCO is commonly discharged into drainage systems, contributing to water contamination. Therefore, this study evaluates the alkaline-assisted co-processing of waste cooking oil with crude and distilled expanded polystyrene (EPS) pyrolysis fractions to obtain liquid products with potential application as fuel blendstock components. Specifically, the work explores the co-valorization of WCO with two aromatic hydrocarbon fractions derived from EPS pyrolysis: crude EPS pyrolysis oil and its distillate fraction. These EPS-derived streams are evaluated as residual hydrocarbon co-feeds for the alkaline-assisted processing of WCO into liquid fuel-like products. The influence of the catalyst loading, WCO-to-EPS-derived fraction mass ratio, and EPS-derived fraction type was analyzed based on the liquid product yield. Furthermore, first-generation vegetable oils were tested under selected conditions to compare their behavior with WCO and assess the applicability of the process to different lipid feedstocks. Finally, the fuel-related properties of the obtained liquid products were evaluated through the density, kinematic viscosity, and heating value, and compared with commercial fuel specifications. The results showed liquid product yields up to 92%, kinematic viscosity values within the range of international fuel specifications under selected conditions, and heating values above 40 MJ/kg. However, the density values indicated limitations for direct use as standalone fuels; therefore, the obtained products should be considered as potential fuel blendstock components requiring further blending and chemical characterization studies.

## 1. Introduction

The continuous human population and industrial development have considerably increased worldwide energy demand. The global total energy consumption was 9938 Mtoe in 2018, out of which oil has a maximum contribution of approximately 41% [1]. In addition, the considerable depletion of fossil fuels (oil, coal, and natural gas) has created energy and environmental challenges with the development of sustainable alternatives to minimize this problem [2].

Additionally, the consumption of cooking oil in households, restaurants, hotels, catering, and food processing industries each day worldwide causes an increased production of waste cooking oil (WCO) [3,4,5]. Most of the WCO is disposed of into a dustbin or drainage system or onto the soil. It is estimated that a liter of oil thrown into water sources can contaminate up to 50000 m^3^ of water [6]; therefore, its disposal creates many environmental issues.

Waste cooking oil comprises a wide range of animal and vegetable oils, including sunflower, palm, rapeseed, soybean, and others, and is generally combined [5]. Those mixed oils are considered highly hazardous wastes because they are degraded triglyceride compounds from animal and vegetable sources that have been heated and exposed to various foods that can cause the denaturation of molecules.

Waste cooking oils are mostly made up of triglycerides, monoglycerides, diglycerides, and free fatty acids in varying amounts (5–20% by weight) produced during frying [7]. Saturated and unsaturated fatty acids are the principal components of triglycerides, which may be employed as starting materials for producing value-added products in various industries [4].

Large amounts of WCO and animal fats are accessible throughout the globe, particularly in wealthy nations. In addition, one of the advantages of WCO is that these fatty waste feedstocks are not competitive with human consumption or agriculture and do not create a food vs. fuel crisis. Moreover, their valorization can also solve environmental issues associated with their disposal [3,8].

Waste cooking oil (WCO) is a complex lipid-based residue whose composition depends on the type of oil, frying conditions, degradation degree, water content, and concentration of free fatty acids (FFAs). These characteristics usually complicate its direct conversion through conventional biodiesel routes, since high FFA and moisture contents may promote hydrolysis, soap formation, and lower product yields during alkaline transesterification. For this reason, WCO commonly requires pretreatment or acid esterification before conventional biodiesel production [9]. Despite these limitations, WCO remains one of the most attractive residual feedstocks for energy and chemical valorization because it is widely available, does not compete with food resources, and its use contributes to reducing environmental impacts associated with improper disposal [10,11]. Current WCO valorization pathways include direct combustion for energy recovery [12], biodiesel synthesis [13,14], catalytic upgrading, and its use as a lipid-rich feedstock for alternative fuel blendstocks. Recent studies on the catalytic valorization of waste-derived oxygenated feedstocks have also emphasized the importance of converting highly oxygenated residues into less oxygenated products with improved compatibility for energy and chemical applications. For example, Coccia et al. reviewed transition-metal-catalyzed routes for glycerol reduction, highlighting that oxygen-rich agro-industrial by-products can be upgraded into lower-oxygen compounds, hydrocarbons, fuels, or chemical building blocks through catalytic strategies [15]. This supports the broader relevance of catalytic and assisted upgrading routes for transforming residual lipid-related streams into value-added products.

Conventional biodiesel production is based on the transesterification of triglycerides with short-chain alcohols, usually methanol or ethanol, producing fatty acid alkyl esters and glycerol under relatively mild conditions [8,16]. However, this reaction requires alcohols with suitable functional groups, controlled feedstock quality, and subsequent purification steps to remove glycerol, catalyst residues, soaps, and unreacted compounds. In contrast, the EPS pyrolysis-derived liquids evaluated in this work are mainly aromatic hydrocarbon mixtures and should not be considered alcohol substitutes in a transesterification reaction. Therefore, the present approach is not framed as conventional biodiesel synthesis, but rather as an alkaline-assisted co-processing strategy in which WCO is combined with EPS-derived pyrolytic fractions to obtain liquid products with potential use as fuel blendstock components.

From a circular economy perspective, this approach addresses two problematic waste streams simultaneously: WCO, which is commonly mismanaged and discharged into drainage systems, and expanded polystyrene waste, which is difficult to recycle mechanically. Although conventional biodiesel production is affected by feedstock cost, alcohol consumption, pretreatment requirements, and product purification [17,18,19], the co-valorization of WCO with EPS pyrolysis fractions offers a different pathway focused on upgrading residual lipid and plastic-derived streams into liquid fuel-like products. In this context, the physicochemical properties of the obtained products, such as the density, viscosity, and heating value, are used as initial indicators of their potential application as fuel blendstocks, while further molecular characterization is necessary to clarify the chemical transformations involved and the final product composition. Accordingly, this study does not claim the formation of conventional biodiesel or fatty acid alkyl esters. Instead, the obtained liquid phase is interpreted as a complex fuel-like mixture whose final composition may include unreacted or transformed lipid-derived compounds, EPS-derived aromatic hydrocarbons, and possible alkaline reaction products. This limitation is explicitly considered when discussing the fuel properties and potential applications of the products.

Another major environmental challenge is the increasing production and final disposal of plastic waste. Plastics are light, flexible, resistant, and have a low-cost production and their use has become an essential role in human society and covers many applications [20]. Annual plastic production was 400.3 million tons in 2022 and is estimated to reach 500 million tons in 2028 [21,22].

However, the natural degradation of plastics can take billions of years because the molecules that constitute them (carbon, hydrogen, nitrogen, and chloride) are linked through solid bonds [23]. In addition, plastics destined for packaging represent 40% of all plastic production, which is problematic due to their short service life [24].

Globally, it is estimated that 55% of discarded plastics end up in a landfill or the environment, 20% are recycled, and 25% are incinerated for energy recovery [25,26,27].

In particular, the impact of the incineration of plastic waste on the environment and human health is of concern. Greenhouse gas emissions from incineration will reach 6.5 Gigatons of CO_2_ equivalent by 2050 [28]. Therefore, incineration should only be used as a last resort and implementing suitable treatment methods for plastic waste in this context is necessary.

An alternative recycling method explored is the pyrolysis process, which converts plastic waste into fuel-like hydrocarbons by transforming large hydrocarbon chains into smaller chains under an inert atmosphere or limited oxygen environment and at high temperatures [29].

Moreover, polystyrene (PS) is heat-resistant and lightweight; it has good strength and durability, making this polymer suitable for various applications such as food packaging, beverages, household appliances, and insulating systems for the construction industry.

One of the beneficial properties of PS pyrolytic oils is their high energy content; nevertheless, it is not recommended to be used directly as fuel due to their high aromatic content. However, blending them with other fuels or biofuels can improve their high heating value.

In the literature survey, a reaction like the one proposed was not found; there are studies on obtaining biofuels by the co-pyrolysis of expanded polystyrene (EPS) with biomass. One example is that of Reshad et al. [30], who studied the co-pyrolysis of EPS with rubber seeds. The study evaluated the effects of the pyrolysis temperature (350−600 °C) and heating rate (10−40 °C·min^−1^) on product distribution (liquid, gas, and biochar). They found that the optimum conditions of the process were 500 °C and a heating rate of 20 °C. The maximum yield of liquid was obtained as (48.25 wt.%) with a calorific value of 32.25 MJ/kg, comparable to mineral fuel. The authors concluded that the co-pyrolysis of rubber seed with waste polystyrene is promising to enhance the bio-oil yield and energy content without changing the pyrolysis condition.

Moreover, Shadangi and Mohanty [31] showed the pyrolysis process to convert biomass and PS waste into fuel, improving the quality of pyrolytic oil with Niger seeds. This paper aims to improve the heating value and other properties in cold environments and, in addition, to reduce the viscosity of the oil. The yield and quality of co-pyrolytic oil were compared with the thermal pyrolytic oil of these seeds. The authors concluded that the co-pyrolysis of waste polystyrene and seed not only enhanced the conversion of seed to product but also significantly affected the co-pyrolytic oil fuel properties. For example, the viscosity of the co-pyrolytic oil was reduced, and a higher calorific value of co-pyrolytic oil was obtained. Finally, Sarmiento et al. [32] reported a gasifying process of EPS and WCO to produce a possible construction material, where morphological, chemical, and mechanical tests were performed. The authors reported that both precursor wastes (EPS and WCO) are significant pollutants when released into the environment, and any process that removes these two waste products from the waste stream benefits society.

The present study explores the co-valorization of waste cooking oil (WCO) and expanded polystyrene (EPS) pyrolysis-derived fractions to obtain liquid products with potential use as fuel blendstock components. Unlike conventional biodiesel production, this approach is not described as transesterification or alcohol substitution, since EPS pyrolytic oil and its distillate are mainly aromatic hydrocarbon mixtures. Instead, the work evaluates an alkaline-assisted co-processing route using WCO, crude EPS pyrolytic oil, and its distillate fraction. The influence of the feedstock type, EPS-derived fraction, catalyst loading, and mass ratio on the liquid product yield was statistically analyzed, and the resulting products were characterized through the density, kinematic viscosity, and heating value.

## 2. Materials and Methods

### 2.1. Materials

The raw material used in this work was EPS waste generated during packaging manufacturing processes and supplied by “Poliespuma del Bajío” manufacturer in Irapuato, Guanajuato, Mexico. This residue, a powder with a nominal density ranging from 8 to 12 kg/m^3^, resulted from the company’s recycling process. As the material came directly from the company, no cleaning or thermal pretreatment was carried out. Since the EPS waste originated from a specific industrial recycling stream, its composition may differ from post-consumer or mixed EPS wastes containing additives, pigments, coatings, or contaminants. Therefore, the reproducibility of the process with EPS from other sources may require previous characterization of the density, contamination level, and additive content.

In addition, commercial soybean, castor, coconut, and palm oils were supplied by traditional markets of Salamanca, Guanajuato.

Moreover, the WCO used in this work was collected from several restaurants in Salamanca, Guanajuato, and was vacuum-filtered with a Whatman™ Grade 1 qualitative filter paper (Cytiva, Marlborough, MA, USA) to retain the suspended solids up to 11 µm to eliminate other elements. Finally, technical-grade potassium hydroxide (KOH) flakes with 98% purity were used as a catalyst and were supplied by Sigma-Aldrich (St. Louis, MO, USA).

### 2.2. Characterization of Raw and Product Oils

The determined properties were the density, viscosity, and heating value following ISO and ASTM standards. For EPS pyrolysis oil, its distillate, and the recovered liquid products, density measurements were made using a glass hydrometer under the ASTM D1298 standard at 20 °C [33]; for WCO, the measurement was made using the ISO 6883 standard [34]. Additionally, a Cannon-Fenske viscometer was used to determine the kinematic viscosity of EPS pyrolysis oil, its distillate, WCO, and the recovered liquid products following the ASTM D445 standard [35]. Moreover, the heating value was determined using an IKA C3000 isoperibolic bomb calorimeter (IKA, Staufen, Germany) under the ASTM D4809 standard [36]. Finally, a Varian 450 GC gas chromatograph (Varian, Waltham, MA, USA) was used to determine the chemical composition of the raw lipid feedstocks and EPS-derived pyrolysis fractions.

### 2.3. Obtaining EPS Pyrolysis Oil (EPSO) and Distilled EPS Pyrolysis Oil (DEPSO)

**Figure 1:**
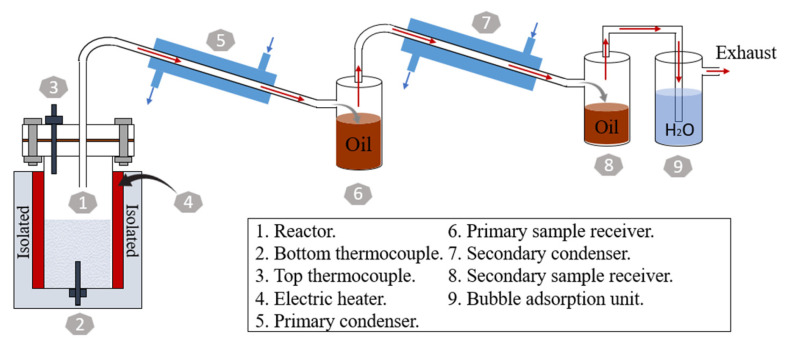
Experimental setup for thermal pyrolysis of EPS waste.

The EPS pyrolysis oil (EPSO) used in the present study was obtained as a liquid hydrocarbon product from the thermal pyrolysis of EPS waste at temperatures ranging from 350 to 400 °C, a range in which high liquid yields have been previously reported [37,38]. Figure 1 shows the experimental setup of the thermal pyrolysis of EPS waste. The process was developed in a stainless-steel semi-batch reactor heated by a band-type resistor with a ceramic core as detailed in previous work. The system was designed and developed by the “CMT-UG” research group, whose Spanish name refers to alternative fuels and internal combustion engines, at the Engineering Division, Irapuato-Salamanca Campus, University of Guanajuato, Mexico [38].

To obtain the distilled EPS pyrolysis oil fraction (DEPSO), the crude EPS pyrolysis oil was subsequently fractionated by atmospheric distillation. The light fraction collected at evaporation temperatures below 160 °C was used as DEPSO.

### 2.4. Experimental Setup

**Figure 2:**
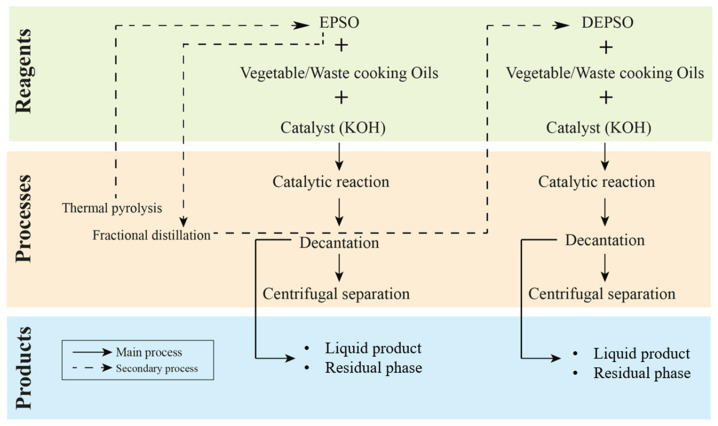
Alkaline-assisted co-processing scheme.

The experimental scheme is shown in Figure 2. The catalytic reaction was carried out in a 250 mL glass reactor coupled to a 50 cm straight condenser to avoid the evaporation of the reagents; heating and stirring were carried out with the help of a stirring hotplate. The magnetic-type agitation reached an average speed of 1000 rpm, and the reaction temperature was kept at 120 °C for 90 min. Subsequently, the products were separated using a 250 mL funnel through density difference. The settling was left to act for 24 h; the two products were separated afterward.

The upper liquid phase was collected and referred to as the liquid fuel-like product, while the remaining phase was considered the residual phase. These operational terms were assigned based on phase separation and visual characteristics; however, they do not imply that the liquid product corresponds to conventional biodiesel. Finally, a centrifugal separation operated at an average speed of 4000 rpm for 10 min was performed to remove catalyst residues and impurities from the recovered liquid product.

### 2.5. Experimental Design

The main objective of this work was to evaluate crude expanded polystyrene pyrolysis oil (EPSO) and its distillate fraction, referred to as distilled EPS pyrolysis oil (DEPSO), as co-processing agents for the valorization of waste cooking oil (WCO) into liquid fuel-like products. The study was not framed as a conventional transesterification reaction or as an alcohol substitution route, since EPSO and DEPSO are mainly aromatic hydrocarbon mixtures. The experiments were divided into two stages. In the first stage, a full factorial design with central points was applied to analyze the influence of the WCO-to-EPS-derived fraction mass ratio, the type of EPS-derived fraction, and catalyst loading on the liquid product yield.

The response variables analyzed were the liquid product yield and the residual phase yield. In the first experimental stage, the lipid feedstock-to-EPS-derived fraction mass ratio, defined as the mass ratio between WCO and either EPS pyrolysis oil (EPSO) or distilled EPS pyrolysis oil (DEPSO), was varied from 0.6 to 1.4. Catalyst loading was evaluated between 1 and 3 wt.%, while the remaining operating conditions are summarized in Table 1. In the second stage, different first-generation vegetable oils were used as reference lipid matrices and processed with EPSO and DEPSO under the selected conditions to compare their conversion behavior with that of WCO. For both stages, the liquid product yield was calculated using Equation (1), where $m_{f}$ is the mass of the liquid product obtained and $m_{T}$ is the total initial mass of the lipid feedstock and EPS-derived fraction used in the process.

(1) $$Yield_{Fuel}(\%)=\frac{m_{f}}{m_{T}}\cdot 100$$

## 3. Results

### 3.1. Characterization of EPS Pyrolysis Oil and Distilled EPS Pyrolysis Oil

Table 2 shows clear differences between EPSO and DEPSO. EPSO presented a higher density and viscosity, with values of 943 kg/m^3^ and 1.43 mm^2^/s, respectively, compared with 896 kg/m^3^ and 0.66 mm^2^/s for DEPSO. This indicates that DEPSO is a lighter and less viscous fraction due to the distillation process.

Both fractions showed similar heating values: 41.65 MJ/kg for EPSO and 42.29 MJ/kg for DEPSO. However, their carbon distribution was markedly different. EPSO contained 55.70 wt.% C7–C10, 26.48 wt.% C11–C14, and 17.79 wt.% C15–C30, while DEPSO was mainly composed of C7–C10 compounds, reaching 99.21 wt.%. Therefore, EPSO is a broader and heavier hydrocarbon mixture, whereas DEPSO is a lighter fraction concentrated almost entirely in low-carbon-range compounds.

From a mechanistic perspective, the alkaline-assisted processing of WCO with EPS-derived aromatic fractions should not be interpreted as conventional transesterification. Under the evaluated conditions, namely 120 °C, 90 min, and KOH catalysis, partial hydrolysis or saponification of triglycerides may occur, leading to the formation of lipid-derived species such as fatty acid salts, glyceride fragments, or unreacted lipid compounds. At the same time, EPSO and DEPSO may mainly contribute aromatic hydrocarbons, including styrene and related compounds, which may remain physically incorporated in the liquid phase or undergo secondary reactions such as oligomerization or polymerization. Since the final liquid product was not characterized by GC–MS, FTIR, or NMR, its molecular composition cannot be conclusively assigned. Therefore, the product is conservatively described as a complex liquid fuel-like mixture or potential fuel blendstock component rather than biodiesel. Consequently, the reported yield should be interpreted as the recovered liquid-phase yield and not as chemical conversion into a defined molecular product.

### 3.2. Vegetable Oil Characterization

Table 3 summarizes the main properties of the vegetable oils and waste cooking oil (WCO) used in this work. The fatty acid profile of WCO was mainly composed of linoleic acid, with 48.36%, followed by a second major fraction of 29.96%, which is consistent with the composition commonly associated with soybean-based cooking oils. The presence of free fatty acids increased as a consequence of repeated heating and contact with food during frying. Although the free fatty acid content remained below 1%, its value was close to the recommended limit for stable processing, suggesting that the WCO composition may influence phase separation, residue formation, and liquid product yield during co-processing with EPS-derived fractions.

### 3.3. Liquid Product Yield from WCO Co-Processing

**Figure 3:**
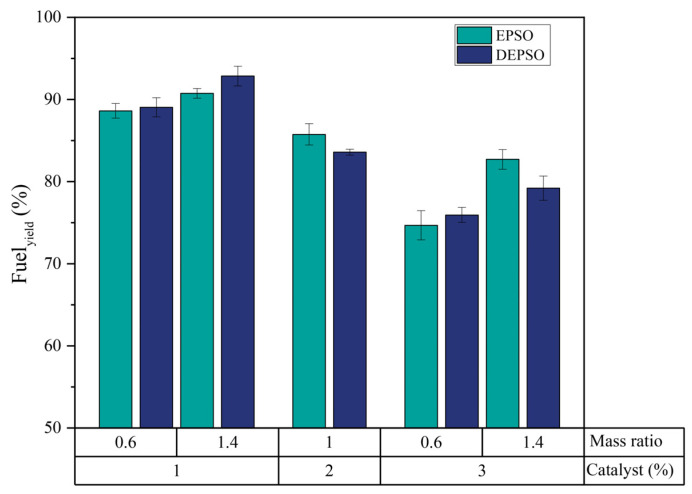
Liquid product yields obtained from WCO co-processing with EPS-derived fractions: EPSO, EPS pyrolysis oil; DEPSO, distilled EPS pyrolysis oil.

The influence of the WCO-to-EPS-derived fraction mass ratio, catalyst loading, and fraction type, namely EPS pyrolysis oil (EPSO) and distilled EPS pyrolysis oil (DEPSO), on the liquid product yield is shown in Figure 3. The results indicate that both EPS-derived fractions allowed high liquid yields, although their performance depended on the operating conditions. For EPSO, the liquid product yield varied from 74.68 to 90.76%, while DEPSO produced yields between 75.95 and 92.86%. The highest yield was obtained using DEPSO at a mass ratio of 1.4 and 1 wt.% catalyst loading. In general, increasing the mass ratio favored liquid product formation, especially at low catalyst loading. Conversely, increasing the catalyst loading tended to decrease the liquid yield for both EPSO and DEPSO, suggesting that lower catalyst concentrations may be sufficient and economically advantageous for the co-processing of WCO with EPS-derived pyrolytic fractions.

### 3.4. Comparison of Fuel-Related Properties with Commercial Fuel Specifications

#### 3.4.1. Density

**Figure 4:**
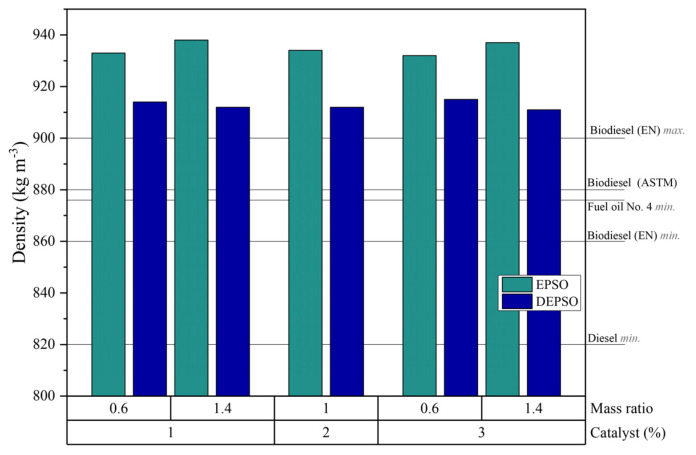
Density of liquid products obtained from WCO co-processing with EPS-derived fractions.

In terms of the fuel properties, density is one of the principal properties used to estimate the quantity of fuel injected by the injection systems to provide proper combustion. Fuel density plays a crucial role in injector nozzle design because it directly affects the engine operation. Moreover, this can directly influence fuel atomization, affecting the engine’s thermal efficiency [39]. The density of biodiesel fuels depends on several factors like the feedstock used, method of biodiesel conversion, and methyl ester profile [39]. Density is one of the relevant parameters that may limit the direct use of fuel-like products when the values fall outside established fuel specifications. Figure 4 compares the density of the liquid products obtained from WCO co-processing with EPS-derived fractions with diesel, biodiesel, and fuel oil specification ranges.

Biodiesel density is commonly specified within the range of 860–900 kg/m^3^, whereas petroleum diesel is generally allowed within 820–860 kg/m^3^. In comparison, conventional biodiesel produced from WCO has been reported with an average density of approximately 878.8 kg/m^3^ [40,41,42,43]. In the present study, the liquid products obtained from WCO co-processing with EPS-derived fractions showed higher density values than those commonly accepted for neat diesel and biodiesel fuels. This behavior can be associated with the aromatic nature and relatively high density of the EPS pyrolysis-derived fractions, particularly crude EPS pyrolysis oil (EPSO). However, the use of distilled EPS pyrolysis oil (DEPSO) resulted in lower density values than EPSO under the evaluated mass ratios and catalyst loadings, which may be attributed to the enrichment of lighter compounds during distillation. Although the obtained liquid products do not fully comply with the density requirements for direct use as standalone fuels, they may be considered as potential fuel blendstock components. Previous studies have shown that blending biofuels with petroleum diesel can reduce the density of the final mixture by up to 5%, especially in blends containing 20% biofuel and 80% diesel [44,45]. Therefore, further blending studies are required to determine suitable incorporation levels and verify compliance with fuel specifications.

#### 3.4.2. Viscosity

Viscosity is one of the vital characteristics of a fuel that signifies the ability of the fuel to flow, playing a significant role in spray atomization and penetration. Furthermore, fuel viscosity is a critical parameter because its value must be low enough to flow through the feed circuits without excessive pressure drops and, in some cases, such as diesel and fuel oil, high enough to meet specific lubricating requirements [46].

**Figure 5:**
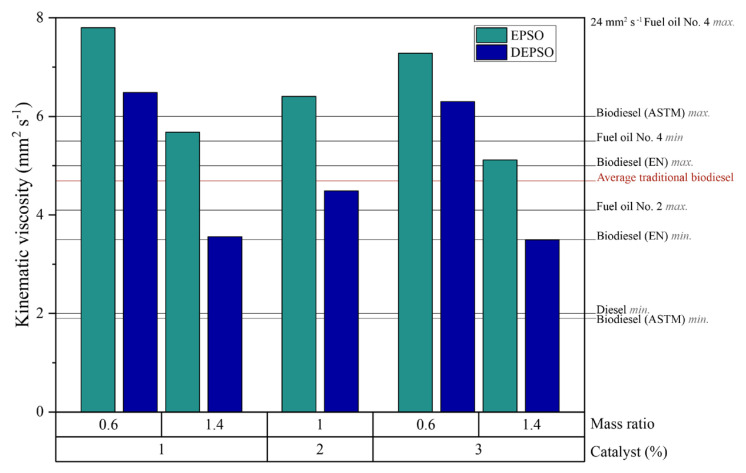
Kinematic viscosity at 40 °C of liquid products obtained from WCO co-processing with EPS-derived fractions.

Since biodiesel possesses a more extensive chemical structure and molecular mass, the viscosity of biodiesel is 10–15 times higher than conventional fossil fuel [46,47]. Higher viscous fuels cause insufficient fuel atomization, reducing thermal efficiency and soot deposits. On the other hand, reduced viscosity leads to finer fuel droplets, which make it easy for the injector to pump fuel into the combustion chamber [39]. Figure 5 compares the kinematic viscosity of the liquid products obtained from WCO co-processing with EPSO and DEPSO with diesel and biodiesel specification ranges.

Allowed kinematic viscosity values for biodiesel lie in the range of 1.9 to 6 mm^2^/s, while specification for diesel is reported to be between 2 and 4.5 mm^2^/s. Traditional WCO biodiesel averages 4.69 mm^2^/s [40,41,42,43]. The kinematic viscosity values of the recovered liquid products were mainly within the ranges established by the evaluated international specifications. The DEPSO-derived products showed that a minimum lipid feedstock-to-DEPSO mass ratio of 1.0:1 was necessary to obtain kinematic viscosity values within the evaluated fuel specification ranges. In contrast, for EPSO-derived products, the 0.6:1 mass ratio did not provide viscosity values within the evaluated specification ranges, even when catalyst loading was increased. Therefore, a minimum mass ratio of 1.4:1 is recommended for EPSO under the tested conditions.

#### 3.4.3. Heating Value

**Figure 6:**
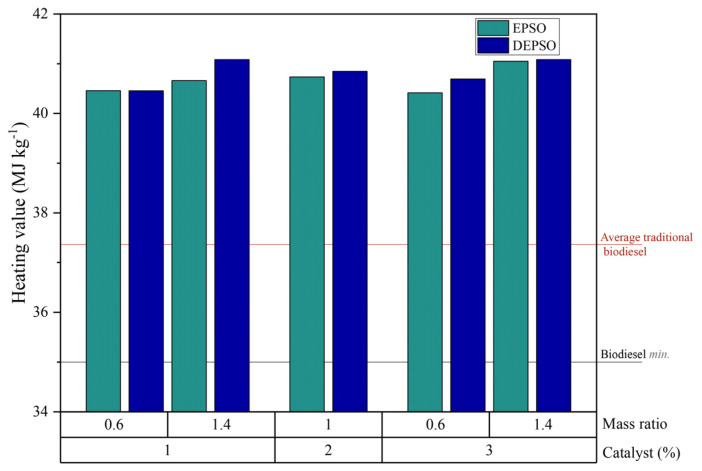
Heating value of liquid products obtained from WCO co-processing with EPS-derived fractions.

The energy of a fuel is expressed by its heating value (HV), which is defined as the magnitude of the heat of the reaction at a constant pressure or volume at a standard temperature, usually 25 °C, for the complete combustion of a unit mass of fuel [48]. The HV of the biodiesel fuel is lower than that of conventional diesel fuel. Even though the heating value is not specified in the ASTM D6751 and EN 14214 standards [49,50], a minimum value of 35 MJ/kg has been reported in EN 14213 (biodiesel for heating purposes) [51,52]. Regarding the fuel energy content, it has been indicated that blending PS pyrolytic oil with biomass or conducting co-pyrolysis of both feedstocks increased the heating value of the biofuels [30,31,53,54,55,56,57]. Figure 6 shows the heating value of the liquid products obtained from WCO co-processing with EPS-derived fractions, and they are compared with the EN 14214 specifications and the average heating value of a traditional WCO biodiesel [40,41,42,43,44,45,58,59,60,61,62,63,64,65,66,67,68,69]. The use of EPS-derived fractions contributed to high heating values in the recovered liquid products, with values above 40 MJ/kg.

### 3.5. Influence of Raw Material on Fuel Production

To assess the applicability of the co-processing approach to different lipid feedstocks, four first-generation vegetable oils were evaluated: soybean, castor, coconut, and palm oil. The mildest operating conditions previously tested with WCO were selected as follows: a mass ratio of 0.6 and a catalyst loading of 1 wt.%. These conditions were not selected as the maximum yield condition, but rather as a mild compromise condition with lower catalyst loading and lower EPS-derived fraction demand. Although the highest WCO yield was obtained at a 1.4:1 mass ratio, the 0.6:1 condition was selected to evaluate whether acceptable liquid yields could still be obtained under reduced reagent requirements. The tests were performed using both EPS-derived fractions: EPS pyrolysis oil (EPSO) and distilled EPS pyrolysis oil (DEPSO).

**Figure 7:**
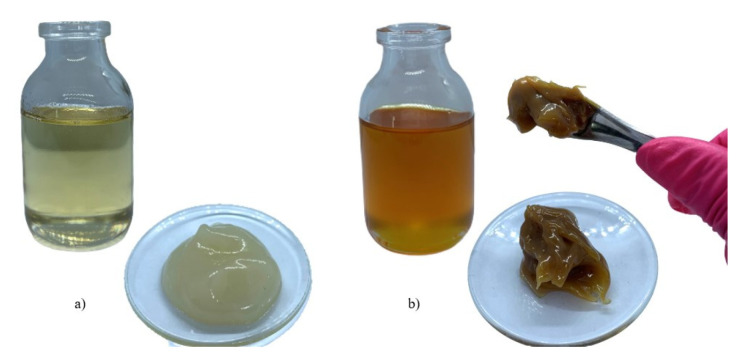
Liquid products obtained from coconut oil co-processed with EPS-derived fractions: (**a**) DEPSO and (**b**) EPSO.

Figure 7 shows the visual appearance of the liquid products obtained from the different vegetable oils, with particular emphasis on coconut oil processed with DEPSO, which produced a lighter product, and with EPSO, which produced a darker product. In general, the products obtained with EPSO showed an amber-to-dark hue, whereas those obtained with DEPSO presented a lighter and more translucent appearance. This difference may be associated with the composition of each EPS-derived fraction, since DEPSO is expected to contain a higher proportion of lighter aromatic compounds. Additionally, the residual phase obtained with EPSO appeared denser than that obtained with DEPSO, suggesting a greater contribution of heavier compounds from the crude pyrolytic oil fraction.

**Figure 8:**
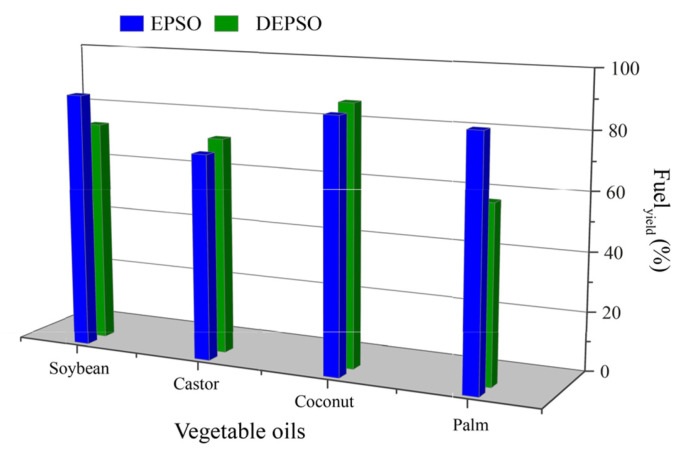
Liquid product yields from vegetable oils co-processed with EPS-derived fractions: EPSO and DEPSO.

Figure 8 shows the liquid product yields obtained from the co-processing of four first-generation vegetable oils with the EPS-derived fractions. The highest yield was obtained with soybean oil using EPS pyrolysis oil (EPSO), reaching 88.8%, followed by coconut oil using distilled EPS pyrolysis oil (DEPSO), with a yield of 87.3%. Coconut oil showed favorable behavior with both EPS-derived fractions, also reaching a high liquid yield of 86.2% when processed with EPSO. In contrast, the lowest yield, below 60%, was obtained when palm oil was processed with EPSO. These results suggest that the lipid feedstock type and the EPS-derived fraction strongly influence liquid product formation. However, additional physicochemical and molecular characterization is required to establish more accurate conclusions regarding the composition and potential application of the obtained products.

The differences observed between WCO and first-generation vegetable oils suggest that liquid product formation is not governed exclusively by triglyceride conversion. WCO contains degraded lipid compounds, free fatty acids, oxidation products, water traces, and other frying-derived impurities that may influence phase separation, alkaline reactions, and the incorporation of EPS-derived hydrocarbons into the liquid phase. Therefore, the higher yields observed for WCO under some conditions may be related to its complex composition rather than to triglycerides alone.

## 4. Conclusions and Recommendations

This study evaluated the alkaline-assisted co-processing of waste cooking oil (WCO) with two EPS-derived pyrolysis fractions, EPSO and DEPSO, to obtain liquid fuel-like products with potential application as fuel blendstock components. The process was not interpreted as conventional biodiesel synthesis or as an alcohol substitution route, since EPSO and DEPSO are mainly aromatic hydrocarbon mixtures and do not provide the hydroxyl functionality required for transesterification.

Liquid product yields of up to 92% were obtained, with the highest yield achieved using DEPSO at a WCO-to-DEPSO mass ratio of 1.4:1 and 1 wt.% KOH. In general, increasing the lipid feedstock-to-EPS-derived fraction mass ratio favored liquid product formation, while increasing catalyst loading tended to reduce the liquid product yield under the evaluated conditions.

The obtained liquid products showed heating values above 40 MJ/kg, which indicates a high energy content compared with conventional biodiesel. However, the density values exceeded the usual specification ranges for neat diesel and biodiesel fuels. Therefore, the products should not be presented as standalone fuels at this stage, but rather as potential blendstock components requiring further blending studies with petroleum-derived fuels or other compatible fuel matrices.

Regarding kinematic viscosity, selected EPSO- and DEPSO-derived products showed values within the evaluated fuel specification ranges. For DEPSO-derived products, acceptable viscosity values were obtained from a lipid feedstock-to-DEPSO mass ratio of 1.0:1, whereas EPSO-derived products required a higher mass ratio of 1.4:1 under the tested conditions.

The comparison with first-generation vegetable oils showed that the feedstock composition significantly influences the liquid product yield. The behavior of WCO cannot be attributed only to triglycerides, since degraded lipid compounds, free fatty acids, oxidation products, and frying-derived impurities may also affect alkaline processing and phase separation.

A major limitation of this work is the absence of detailed molecular characterization of the final liquid products. Therefore, future studies should include GC–MS, FTIR, NMR, elemental analysis, acidity, oxidative stability, cold-flow properties, and blending tests to determine the chemical composition, stability, and real applicability of the products as fuel blendstock components.

## Figures and Tables

**Table 1:** Experimental data.

Stage	Parameters	Units	Value/Range
	Fixed parameters		
First	WCO	g	WCO
	Temperature	°C	120
	Agitation speed	rpm	1000
	Time of reaction	minutes	90
	Variable parameters		
	Pyrolytic oil	g	EPSO (Expanded Polystyrene Oil)/DEPSO (Distilled Expanded Polystyrene Oil)
	Lipid feedstock:EPS-derived fraction mass ratio	-	0.6:1, 1:1, 1.4:1
	KOH catalyst loading	wt.%	1, 2, 3
	Fixed parameters		
Second	Temperature	°C	120
	Agitation speed	rpm	1000
	Time of reaction	minutes	90
	Lipid feedstock:EPS-derived fraction mass ratio.	-	0.6:1
	KOH catalyst loading	wt.%	1
	Variable parameters		
	Vegetable oil	g	Soybean, Castor, Coconut, Palm, WCO
	Pyrolytic oil	g	EPSO (Expanded Polystyrene Oil)/DEPSO (Distilled Expanded Polystyrene Oil)

**Table 2:** Physical properties of EPSO and DEPSO.

Property	Unit	EPSO	DEPSO
Density	kg/m^3^	943	896
Kinematic viscosity at 40 °C	mm^2^/s	1.43	0.66
Heating value	MJ/kg	41.65	42.29
Carbon content	wt.%		
C_7_–C_10_		55.70	99.21
C_11_–C_14_		26.48	0.70
C_15_–C_30_		17.79	0.10

**Table 3:** Properties of experimental vegetable and waste cooking oils.

Vegetable Oil	Density @ 15 °C [kg/m^3^]	Viscosity @ 40 °C [mm^2^/s]	Free Fatty Acids [%wt.]	Molecular Weight [g/mol]
Soybean	918	32.57	0.07	928.3
Castor	965	231.22	<0.50	926.0
Coconut	928	89.51	0.24	730.4
Palm	921	78.21	0.47	890.1
WCO	925	70.46	0.72	1001.0

## Data Availability

The original contributions presented in this study are included in the article. Further inquiries can be directed to the corresponding author.

## Abbreviations

ASTM	American Society for Testing and Materials
DEPSO	Distillation of the Pyrolysis Oil of Expanded Polystyrene
EN	European Biodiesel Specification
EPS	Expanded Polystyrene Waste
EPSO	Pyrolysis Oil of Expanded Polystyrene
FAME	Fatty Acid Methyl Esters
FFA	Free Fatty Acid
HV	Heating Value
KOH	Potassium Hydroxide
PS	Polystyrene
WCO	Waste Cooking Oil
